# Unraveling complexity: morbidity factors in elderly kidney transplant recipients

**DOI:** 10.1093/ckj/sfae182

**Published:** 2024-06-18

**Authors:** Alexandra Gineste, Paolo Malvezzi, Thomas Jouve, Claire Millet, Lionel Rostaing, Johan Noble

**Affiliations:** Nephrology, Hemodialysis Apheresis and Kidney Transplantation, Department, CHU Grenoble Alpes, Grenoble, France; Nephrology, Hemodialysis Apheresis and Kidney Transplantation, Department, CHU Grenoble Alpes, Grenoble, France; Nephrology, Hemodialysis Apheresis and Kidney Transplantation, Department, CHU Grenoble Alpes, Grenoble, France; Univ. Grenoble Alpes, CNRS, Inserm, CHU Grenoble Alpes, IAB, Grenoble, France; Department of Geriatrics, Voiron, CHU Grenoble Alpes, Grenoble, France; Nephrology, Hemodialysis Apheresis and Kidney Transplantation, Department, CHU Grenoble Alpes, Grenoble, France; Univ. Grenoble Alpes, CNRS, Inserm, CHU Grenoble Alpes, IAB, Grenoble, France; Nephrology, Hemodialysis Apheresis and Kidney Transplantation, Department, CHU Grenoble Alpes, Grenoble, France; Univ. Grenoble Alpes, CNRS, Inserm, CHU Grenoble Alpes, IAB, Grenoble, France

**Keywords:** elderly, frailty, graft loss, kidney transplantation, mortality

## Abstract

**Background:**

The rising prevalence of end-stage renal failure in the elderly has led to an increased number of kidney transplantations in older individuals. While age does not solely determine transplant eligibility, frailty in elderly recipients significantly impacts post-transplant outcomes, particularly within the first year.

**Methods:**

The RETRAITE (REnal TRAnsplantIon ouTcome in Elderly recipients) study, a single-center retrospective cohort study at Grenoble Alpes University Hospital (France), examined kidney transplant recipients aged 70 years and above transplanted between 2015 and 2020. The composite primary endpoint was defined as either of any hospital stay exceeding 40 days, death and/or return to dialysis within the first post-transplant year. The study explored risk factors for recipient and graft survival, rejection, hospitalizations over 40 days, and severe infections during the initial post-transplant year.

**Results:**

Over six years, 149 patients aged 70 years or older received transplants. Eleven patients died, and seven returned to dialysis within the first year, corresponding to a 1-year graft survival rate of 87.9%. At 1 year, 49 patients (33%) met the composite endpoint. There was a significant association between the composite endpoint and curative anticoagulation [odds ratio (OR) 5.20; *P* < .001], peripheral arteriopathy (OR 3.14; *P* < .001) and delayed graft function (OR 8.24; *P* < .001). This cohort then was merged with a cohort of 150 younger kidney transplanted patients and we confirmed these results. Time on dialysis, prolonged cold ischemia and donor age contributed to higher morbidity and mortality. Conversely, preemptive and living donor transplants were associated with lower morbidity and mortality.

**Conclusions:**

In this cohort aged over 70 years, age alone did not statistically correlate with increased morbidity and mortality. Variables related to grafts and donors, especially curative anticoagulation, were linked to poorer outcomes, emphasizing the favorable impact of preemptive and living donor transplants on morbidity and mortality in elderly patients.

KEY LEARNING POINTS **What was known:**

- The prevalence of chronic renal failure is rising in the elderly, with kidney transplant recipients generally experiencing improved outcomes.
- Frailty in elderly kidney transplant patients is associated with increased complications and mortality.
- Frailty screening tools are limited, and there are no established guidelines on frailty thresholds for transplantation in chronic kidney disease.

**This study adds:**

- Kidney transplantation in elderly patients (70 and above) can lead to excellent outcomes at 1 year, with high recipient and graft survival rates.
- Factors such as preemptive transplants and living donor transplants are associated with lower morbidity and mortality.
- Curative anticoagulation in elderly transplant recipients is a significant risk factor for adverse outcomes, including bleeding complications and fatalities.

**Potential impact:**

1. The findings emphasize the positive outcomes of kidney transplantation in the elderly, guiding clinicians in patient selection.
2. Insights into factors influencing morbidity and mortality, particularly the impact of anticoagulation, can inform pre-transplant assessments and post-transplant care.
3. This study contributes to refining guidelines for managing elderly patients in kidney transplantation, influencing clinical practice and potentially improving outcomes.

## INTRODUCTION

Chronic renal failure is a prevalent condition, impacting one-third of the global population aged 65 years and above [1]. The rising numbers of individuals undergoing dialysis or awaiting kidney transplants pose a significant public health challenge due to the associated costs of renal replacement therapies [2]. Existing literature suggests that kidney transplant recipients generally experience prolonged life expectancy, improved quality of life and reduced societal costs compared with those on waiting lists [3–5]. However, studies highlight a notable increase in post-transplant mortality among patients aged over 70 years, indicating a specific vulnerability in this demographic [6, 7]. Elderly patients exhibit substantial heterogeneity, often presenting with various comorbidities and frailties. Frailty, a prevalent concern in elderly individuals with chronic kidney failure, has been linked to delayed graft function, increased perioperative complications and early rehospitalization, and a 2-fold higher risk of post-transplant death [8–10].

Despite the availability of numerous frailty screening tools, some lack comprehensiveness, while others, though more comprehensive, pose challenges for routine clinical use. Moreover, frailty is a dynamic process necessitating periodic reassessment during medical follow-up [11]. Currently, frailty screenings tools for patients with chronic kidney disease are limited, and there are no established scientific guidelines determining the threshold of frailty at which transplantation should be contraindicated.

In response to these gaps in knowledge, we conducted a retrospective study to identify predictive factors associated with heightened morbidity and mortality during the initial year post-transplant in elderly patients with chronic kidney failure.

## MATERIALS AND METHODS

### Study design

In this study, we retrospectively included all patients aged 70 years and above who received a kidney transplant at Grenoble-Alpes University Hospital, France, from 1 January 2015 to 31 December 2020. We excluded ABO and HLA incompatible transplantations, to focus on patients receiving standard immunosuppression. As a control group, we included patients aged between 60 and 70 years who underwent kidney transplant surgery during the same timeframe at a 1:1 ratio.

The immunosuppression regimen comprised induction therapy with anti-thymoglobulin (0.5–1 mg/kg over 5 days, depending on the immunological risk) and a conventional maintenance immunosuppressive protocol involving tacrolimus with mycophenolate or everolimus. Steroids were systematically tapered and discontinued by the third month after transplantation, except for cases involving immunoglobulin A nephropathy, a history of acute rejection or the presence of donor-specific antibodies.

### Endpoints of the study

The primary endpoint of our study was a composite outcome within the initial year post-transplant, with an occurrence of any of the three following events: prolonged hospital stays exceeding 40 days, death or return to dialysis. The 40 days cutoff for defining “extended” hospitalization stays was chosen as the last quartile of hospital stay lengths within our cohort.

Data collection included demographic characteristics of patients, including cardiovascular risk factors (smoking based on medical record, high blood pressure, dyslipidemia, diabetes and use of insulin) and cardiovascular events (peripheral arteriopathy, acute coronaropathy and chronic heart failure, stroke or transient ischemic attack, and the use of anticoagulation) and characteristics of the kidney transplantation: induction therapy, maintenance immunosuppression, cold ischemia time, donor characteristics and time on dialysis. Delayed graft function was defined as the need of renal replacement over the first week after kidney transplantation.

### Data collection and ethical considerations

The data for this study were extracted from medical records at the Grenoble-Alpes University Hospital. Clinical data collection adhered to a comprehensive clinical database, ensuring consistency with information found in the source documents. To uphold confidentiality and privacy standards, all collected data underwent anonymization before analysis. Ethical considerations were prioritized throughout the data collection process to uphold the rights and privacy of the participants.

All patients signed an informed consent form. All medical data were collected from our database [CNIL (French National committee for data protection) approval number 1987785v0].

### Statistical analysis

Descriptive analyses presented quantitative data as means with standard deviations or medians with interquartile ranges, while categorical parameters were expressed as absolute numbers and relative percentages.

An initial univariate statistical analysis examined both the primary and secondary endpoints. Categorical data were compared using the Chi-squared test, and quantitative data were assessed using the Kruskal–Wallis test based on distribution. All factors significantly associated with the endpoint occurrence were also included in a logistic regression analysis. Results were reported as OR with corresponding 95% confidence intervals (95% CI). Kaplan–Meier survival analyses were conducted for the primary composite endpoint, defined as either a hospital stay exceeding 40 days, death within the first year or a return to dialysis post-transplantation. The same method was applied to assess raw survival and kidney survival. All statistical tests adhered to a two-sided 0.05 level of significance, with a *P*-value considered significant below .05. The statistical analyses were conducted using the R software version 4.2.2.

## RESULTS

### Population characteristics

Between January 2015 and December 2020, 149 patients aged 70 years and above received a kidney transplant at Grenoble University Hospital and were included in the analysis. Baseline characteristics and cardiovascular diseases history of the study population is reported in Table 1. Mean (± standard deviation) age of the study population was 74.9 ± 3.5 years. Overall, 120 patients (88%) were on dialysis before transplantation (73% on hemodialysis and 15% on peritoneal dialysis). Median (interquartile range) time on dialysis was 32 (13–51) months. It was the first kidney transplantation for 133 (89.2%) patients and 25 (16.8%) received the kidney from a living donor. Mean donor age was 72.1 ± 10 years. Mean cold ischemia time was 740 ± 387 min and delayed graft function occurred in 26 (17.7%) patients.

**Table 1:** Baseline characteristics of the population.

	Kidney transplant recipients aged over 70 years (*n* = 149)
Male gender, *n* (%)	105 (70)
Age at transplantation, *n* (%)	
70–74 years	71 (48)
75–79 years	59 (40)
>79 years	19 (12)
High blood pressure, *n* (%)	138 (93)
Dyslipidemia, *n* (%)	88 (59)
Smoking, *n* (%)	77 (52)
Diabetes, *n* (%)	51 (34)
Insulin-treated, *n* (%)	33 (22)
Coronary heart disease, *n* (%)	40 (27)
Anticoagulation, *n* (%)	35 (25)
Peripheral arteriopathy, *n* (%)	23 (15)
With surgical intervention, *n* (%)	11 (7)
Chronic respiratory disease, *n* (%)	34 (23)
Stroke or transient ischemic attack, *n* (%)	15 (10)
Chronic heart failure, *n* (%)	8 (5)

The immunosuppressive induction treatment was based on anti-thymoglobulin in 134 (90%) patients and interleukin-2 receptor blocker (basiliximab 20 mg at Day 0 and Day 4 post-transplant) in 14 (10%) patients. Maintenance immunosuppression comprised tacrolimus plus mycophenolate mofetil in 119 (80%) patients and tacrolimus plus everolimus for the others.

In the control group, comprising 150 patients, the mean age was 64.3 ± 2.8 years. The proportion of females was significantly higher compared with the study group (42.7% versus 29.5%, *P* = .018), and there was a lower incidence of hypertension history (84.5% versus 92.2%, *P* = .027). However, there were no statistically significant differences observed in terms of diabetes rate, peripheral arteritis, anticoagulation use, preemptive kidney transplantation, cold ischemia time or delayed graft function (Supplementary data, Table S1).

### Primary composite outcome

At 1-year post-transplantation, 49 patients of the study cohort (33%) reached the composite endpoint as defined by prolonged hospital stays exceeding 40 days (37 patients), death (11 patients) and/or end-stage graft failure (7 patients). Univariate analysis revealed significant associations between the composite endpoint and various factors, including the presence of curative anticoagulation (*P* < .001), peripheral arteriopathy disease in the recipient (*P* = .032), dialysis vintage (*P* = .014), donor's advanced age (*P* = .025), cold ischemia time (*P* = .019) and delayed graft function (*P* < .001). Conversely, preemptive transplantation (*P* = .026), living donor transplantation (*P* = .002) and induction with basiliximab (*P* = .038) appeared to be statistically protective factors (Table 2).

**Table 2:** Unadjusted correlations with the composite endpoint (defined as cumulated hospitalization >40 days or death or end-stage graft failure) during the first year post-transplantation.

	No criteria of the composite outcome (*N* = 100)	Cumulated hospitalization >40 days or death or end-stage graft failure (*N* = 49)	*P*-value
Age at transplantation, years	75.0 ± 3.6	74.9 ± 3.5	.964
Male gender, *n* (%)	70 (70)	35 (71.4)	.857
High blood pressure, *n* (%)	93 (93)	45 (91.8)	.799
Dyslipidemia, *n* (%)	59 (59)	29 (59.2)	.983
Smoking, *n* (%)	51 (51)	26 (53.1)	.813
Diabetes, *n* (%)			
Insulin-treated, *n* (%)	30 (30)	21 (42.9)	.120
Coronary heart disease, *n* (%)	23 (23)	17 (34.7)	.130
Anticoagulation, *n* (%)	**15 (15)**	**22 (44.9)**	**<.001**
Peripheral arteriopathy, *n* (%)	**11 (11)**	**12 (24.5)**	**.032**
With surgical intervention, *n* (%)	5 (5)	6 (12.2)	.112
Chronic respiratory disease, *n* (%)	19 (19)	15 (30.6)	.113
Stroke or transient ischemic attack, *n* (%)	10 (10)	5 (10.2)	.969
Chronic heart failure, *n* (%)	4 (4)	4 (8.2)	.290
Preemptive transplantation, *n* (%)	**17 (17)**	**2 (4.1)**	**.026**
Duration time on dialysis, months	**29** (**7.8–47**)	**36.0** (**25–56**)	**.014**
Cold ischemia time, min	**765** (**343–978**)	**886** (**725–1030**)	**.019**
Donor age, years	**70.9 ± 11**	**74.6 ± 8**	**.025**
Living donation, *n* (%)	**24 (24)**	**1 (2)**	**.002**
Delayed graft function, *n* (%)	**8 (8)**	**18 (38.3)**	**<.001**
Induction therapy, *n* (%)			
Anti-thymoglobulin	**87 (87)**	**47 (95.9**)	**.038**
Basiliximab	**13 (13)**	**1 (2%)**	

Data are presented as *n* (%), mean ± standard deviation or median (interquartile range).

Bold values are statistically significant comparison between the two groups.

In the multivariate analysis, only the presence of anticoagulation (OR 5.20, 95% CI 2.05–14.08; *P* < .001), peripheral arteriopathy disease (OR 3.14, 95% CI 1.05–9.69; *P* < .001) and delayed graft function (OR 8.24, 95% CI 2.87–26.57; *P* < .001) were independently and statistically associated with the composite endpoint (Table 3).

**Table 3:** Multivariate adjusted correlations with the composite endpoint during the first year post-transplantation.

	OR	95% CI	*P*-value
Anticoagulation	**5.20**	**2.05–14.08**	**<.001**
Peripheral arteriopathy	**3.14**	**1.05–9.69**	**.041**
Dialysis before transplant	2.05	0.33–17.69	.462
Time in dialysis before transplantation	0.99	0.97–1.01	.420
Cold ischemia time	0.99	0.99–1.00	.657
Donor age	1.02	0.98–1.07	.276
Deceased donor	4.26	0.42–103.67	.265
Delayed graft function	**8.24**	**2.86–26.56**	**<.001**
Basiliximab induction	0.22	0.01–1.90	.207

Bold values are statistically significant correlations.

The younger control cohort exhibited a significantly lower rate of reaching the composite endpoint compared with the study cohort (16.7%, *P* = .001). Specifically, among them, 18 patients experienced prolonged hospitalization lasting over 40 days, 4 patients died within the first-year post-transplant and 5 patients encountered graft loss. Subsequently, we expanded our analysis to incorporate the younger patients into a larger cohort (*n* = 299) to validate our findings. In the multivariate analysis, curative anticoagulation and delayed graft function remained significantly correlated with the composite endpoint (OR 4.70, 95% CI 2.27–9.97; *P* < .001 and OR 9.32, 95% CI 4.0–2.3; *P* < .001, respectively). However, peripheral arteriopathy ceased to exhibit a significant association with the composite endpoint. Notably, within this comprehensive cohort, older donor age was identified as a factor linked to the composite endpoint (OR 1.05, 95% CI 1.02–1.09; *P* = .002). These findings are summarized in Supplementary data, Table S2.

### Mortality analysis in post-transplant period

In this study, we recorded 11 deaths (7% of the cohort) within the first year following transplantation. In the univariate analysis, we observed statistical associations between the first-year mortality and the utilization of curative anticoagulation (*P* = .002), dialysis vintage (*P* = .032) and the history of chronic heart failure (*P* = .050). In the subsequent multivariate analysis, only the presence of anticoagulation emerged as a significant factor associated with an increase in recipient mortality, with an OR of 4.56 (95% CI 1.19–19.33; *P* < .001) (Table 4). Upon incorporating the younger cohort into the analysis, anticoagulation remained associated with a heightened risk of all-cause mortality (OR 5.2, 95% CI 1.7–16.7; *P* = .003). Interestingly, recipient age by itself was not associated with first-year mortality in our cohort: 2.7% in the 60- to 70-year-old cohort versus 7% in the study cohort (*P* = .0072).

**Table 4:** Unadjusted and adjusted (multivariate Cox analysis) correlation with first-year mortality.

	Death within the first-year post transplantation (*n* = 11)	Univariate *P*-value	Multivariate OR (95% CI)	Multivariate *P*-value
Age at transplantation, years, mean ± SD	73.8 ± 3.6	.865		
Male gender, *n* (%)	8 (72.7)	.292		
High blood pressure, *n* (%)	10 (90.9)	.833		
Dyslipidemia, *n* (%)	5 (45.5)	.340		
Smoking, *n* (%)	5 (45.5)	.668		
Diabetes, *n* (%)	6 (54.5)	.140		
Insulin-treated, *n* (%)	5 (45.5)	.053		
Coronary heart disease, *n* (%)	3 (27.3)	.974		
Anticoagulation, *n* (%)	**7 (63.6**)	**.002**	**4.56 (1.19–19.33)**	**<.001**
Peripheral arteriopathy, *n* (%)	1 (9.1)	.545		
With surgical intervention, *n* (%)	0 (0.0)	.331		
Chronic respiratory disease, *n* (%)	5 (45.5)	.063		
Stroke or transient ischemic attack, *n* (%)	1 (9.1)	.911		
Chronic heart failure, *n* (%)	**2 (18.2**)	**.050**	0.02 (0.01–0.07)	.284
Preemptive transplantation, *n* (%)	0 (0.0)	.312		
Duration time on the dialysis, months, median (IQR)	55.0 (29.5–64.5)	**.032**	1.01 (0.99–1.04)	.200
Cold ischemia time, min, mean ± SD	885.0 ± 342	.122		
Donor age, years, mean ± SD	73.0 ± 7	.776		
Living donation, *n* (%)	0 (0.0)	.122		
Delayed graft function, *n* (%)	1 (9.1)	.478		
Induction therapy, *n* (%)				
Anti-thymoglobulin	11 (100.0)	.964		
Basiliximab	0 (0)			

SD, standard deviation; IQR, interquartile range.

Bold values are statistically significant correlations.

**Figure 1:**
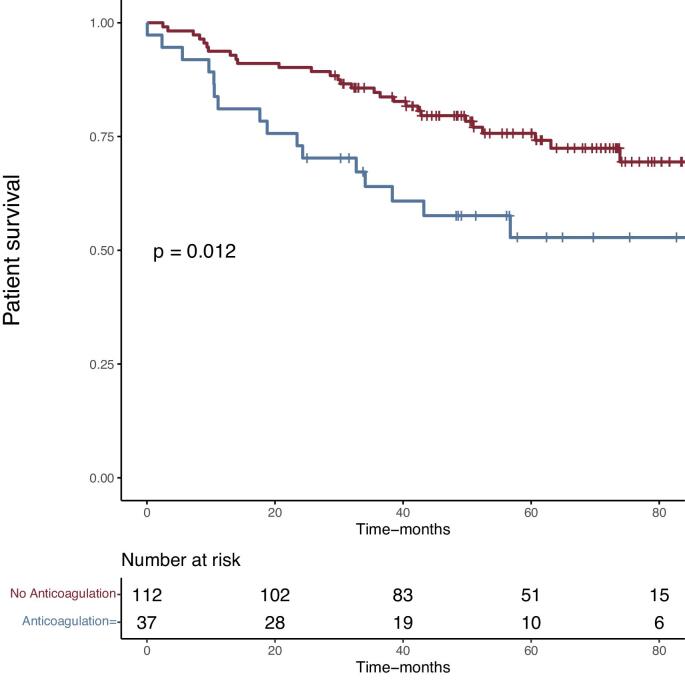
Comparison of the cumulative incidence of patient's survival after kidney transplantation in 149 recipients over 70 years old with and without anticoagulation treatment. Kaplan–Meier plots. *P*-values were calculated with the log-rank test.

**Figure 2:**
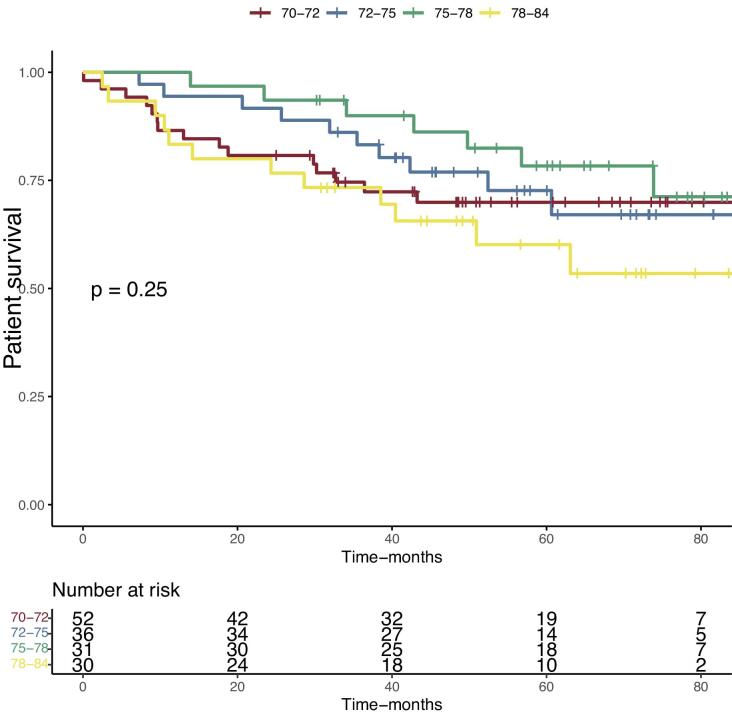
Comparison of the cumulative incidence of patient's survival after kidney transplantation in 149 recipients over 70 years old separated in four groups according to quartile recipient age. Kaplan–Meier plots. *P*-values were calculated with the log-rank test.

**Figure 3:**
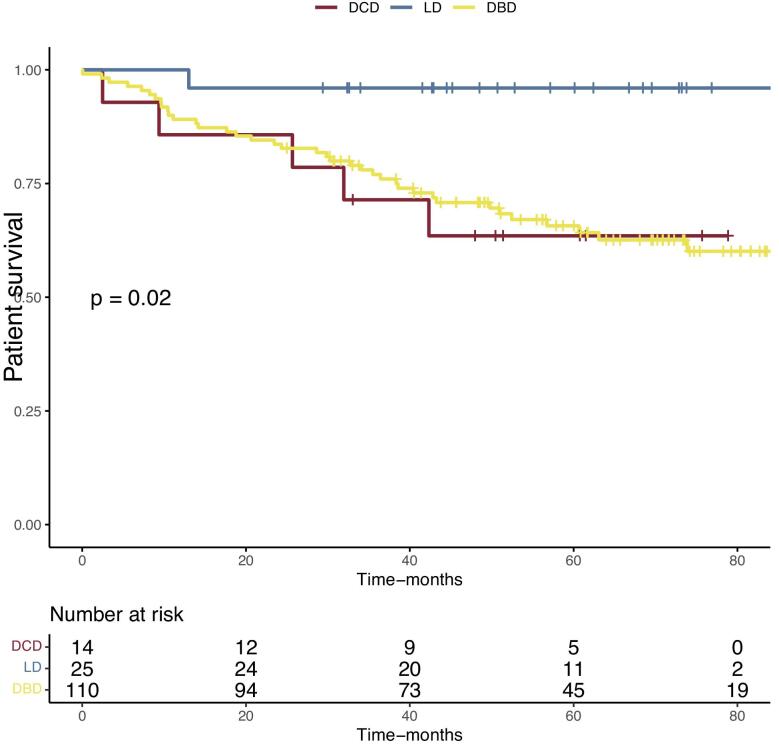
Comparison of the cumulative incidence of patient's survival after kidney transplantation in 149 recipients over 70 years old according to donor, i.e. living donor (LD), donation after brain death (DBD) and donation after circulatory death (DCD). Kaplan–Meier plots. *P*-values were calculated with the log-rank test.

We then assessed the mortality at last follow-up. After a mean follow-up of 51.4 ± 25 months, 46 (30.9%) patients were dead. Kaplan–Meier survival analyses were performed. Survival curves based on anticoagulation confirmed the increased mortality in the curative anticoagulation group of the study cohort (Fig. 1) and in the global cohort included the younger patients (Supplementary data, Fig. S1). We then separated the cohort based on recipient`s age into quartiles and performed Kaplan–Meier survival analysis. Despite a tendency to a higher mortality in the older group (78–84 years old), the overall survival difference between the four groups was not statistically different (Fig. 2). Patient survival at last follow-up was 96% in the living donor group (76 ± 3.3 months, after a median follow-up of 54 months post-transplant), 63.6% in the donation after brain death group (75 ± 3.6 months, after a mean follow of 51 months post-transplantation) and 64.3% in the donation after circulatory death (74.8 ± 3.0 months, after a median follow-up of 46 months post-transplant) (*P* = .02) (Fig. 3).

### Kidney graft survival analysis

Regarding graft outcomes, this investigation revealed that within the first year, seven patients (5% of the total cohort) experienced end-stage kidney graft failure. In univariate analyses, delayed graft function was the main driver of death-censored graft survival (*P* < .001). Notably, 26 patients (17% of the cohort) experienced delayed graft function. Delayed graft function emerged as a significant factor associated with lower graft survival during the first post-transplant year (OR 45.5, 95% CI 6.99–899.57; *P* < .001) in the study cohort alone and combined with the control cohort (Supplementary data, Table S3). In our cohort, six biopsy proven rejections occurred within the first year, i.e. 4%. Two were antibody-mediated and four were T-cell-mediated rejections.

### Cumulative hospitalization analysis

In this cohort, the mean cumulative length of hospital-stay for the whole study population within the first year amounted to a median of 23 (14–40) days. Most of the hospitalizations during the first year were due to infections and it concerned 46 patients (30.8%). In the sub-population of patients that underwent the primary composite outcome, median length of hospital stay was 55 (43–73) days. Univariate analysis revealed statistically significant associations between prolonged hospitalization (>40 days) and the presence of anticoagulation therapy (*P* = .003), peripheral artery disease (PAD) (*P* = .006), arteriopathy requiring surgical intervention (*P* = .018), donor age (*P* = .014), cold ischemia time (*P* = .027) and delayed graft function (*P* < .001) (Supplementary data, Table S4). Conversely, preemptive kidney transplantation (*P* = .035) and living-donor transplants (*P* = .024) were associated with shorter hospital stays. In the multivariate analysis, only anticoagulation therapy (OR 2.9, 95% CI 1.1–7.6; *P* = .027), peripheral arteriopathy (OR 4.1, 95% CI 1.3–12.4; *P* = 0.011) and delayed graft function (OR 6.37, 95% CI 2.27–19.11; *P* < .001) demonstrated a significant association with prolonged hospitalization during the initial post-transplant year (Supplementary data, Table S4). These findings were confirmed when merging the study cohort with the control cohort. Additionally, in this combined analysis, recipient age emerged as significantly associated with prolonged hospitalization during the first year (OR 1.06, 95% CI 1.02–1.11; *P* = .0013)].

### Anticoagulation therapy and complications

Sub-analysis within the anticoagulation cohort was conducted in response to the observed statistical associations between therapeutic anticoagulation and the identified morbidity–mortality endpoints. Supplementary data and detailed information regarding hospitalizations during the initial post-transplant year were subsequently gathered for the 35 patients receiving curative anticoagulation.

Among this subgroup, the anticoagulation regimen primarily consisted of antivitamin K, predominantly prescribed for atrial fibrillation management (77%). Three (9.4%) of these patients returned to dialysis within the first year post-transplant compared with 3.6% observed in the cohort of patients without anticoagulation therapy (*P* = .365). Significantly, there were seven deaths (20%) in the anticoagulation group, versus four deaths (3.6%) in the cohort of patients without anticoagulation therapy (OR 6.19, 95% CI 1.714.7; *P* = 0.005). Hemorrhagic complications, defined by the occurrence of bleeding and/or the presence of a hematoma, were noted in 40% of the anticoagulation subgroup. Additionally, 20% of the patients receiving anticoagulation therapy experienced further surgery, and 37% required blood transfusions. The comprehensive findings of this sub-analysis are detailed in Table 5.

**Table 5:** Indications of anticoagulation and complications post-transplantation in 35 KTx recipients receiving anticoagulation therapy.

	KTx recipients receiving anti-vitamin K therapy (*N* = 35)
Indication, *n* (%)	
Atrial fibrillation	27 (77)
VTE disease	3 (8.6)
Aortic valve replacement	2 (5.7)
Genetic disease	1 (2.8)
Embolism cardiopathy	1 (2.8)
Unknown	1 (2.8)
Complications, *n* (%)	
Blood transfusion post-KTx	13 (37)
Hemorrhagic complication	14 (40)
Return to surgery post-KTx	7 (20)
1st year death	7 (20)
1st year end-stage kidney transplant failure	3 (9)

KTx, kidney transplantation; VTE, vascular thrombo-embolic.

## DISCUSSION

In this study encompassing 149 patients aged 70 years and above who underwent kidney transplantation we observed that 33% of patients reached the composite endpoint at 1-year post-transplantation, defined as prolonged hospital stays, death and/or end-stage graft failure. In the multivariate analysis, the presence of anticoagulation, PAD and delayed graft function remained independently and statistically associated with the composite endpoint. These findings underscore the significance of these factors in assessing the prognosis of elderly patients undergoing kidney transplantation.

In the context of an aging population, kidney transplantations are increasingly performed on elderly recipients and donors. This study stands out for specifically investigating morbidity through a composite endpoint that considers cumulative hospitalization days, departing from the conventional focus on mortality and/or graft survival. This chosen endpoint is particularly relevant in the elderly population, where prolonged and/or repeated hospitalization can have severe consequences. The study's follow-up period spans the first year post-transplant, a critical phase with a heightened risk of postoperative complications in patients with numerous frailties [11, 12].

Our investigation provides an objective assessment of favorable outcomes at 1 year for transplant recipients aged 70 years and older. Within this cohort, we achieved a remarkable 93% recipient survival and 88% graft survival at the 1-year mark. These results align with findings by Karim *et al.*, demonstrating a first-year post-transplant mortality of 11.9% in recipients aged 70–79 years and 9.1% in those aged over 80 years [13]. Furthermore, our study reveals that, beyond the age of 70 years, the recipient's age itself is not statistically associated with higher mortality during the first-year post-transplant but was associated with the composite endpoint of death, length of hospitalization and end-stage renal disease within the first-year post-transplantation. Instead, the primary influencing factors are the recipient's comorbidities, notably the presence of curative anticoagulation and/or arteriopathy, the type of donation and graft quality. To correct for the effect of recipient age, we verified these results by conducting the analysis including a control group comprising patients transplanted during the same period and aged between 60 and 70 years old.

The type of transplant emerges as a crucial determinant, with preemptive and living-donor transplants statistically linked to lower morbidity and mortality. Basiliximab induction stands out as a positive prognostic factor, likely attributed to its reserved use in our center for living donor transplants. Conversely, prolonged cold ischemia duration, advanced donor age, extended waiting time on dialysis and delayed graft function recovery were associated with increased morbidity and mortality [14]. Notably, univariate analysis reveals a statistical association between prolonged waiting time on the transplant list and death, a finding supported by prior studies highlighting the worsening of frailty and heightened death risk during dialysis [10].

Concerning anticoagulation for arrhythmia, current recommendations stress the importance of evaluating risks using CHA2DS2-VASc and HAS-BLED (Hypertension, Abnormal Renal/Liver Function, Stroke, Bleeding History or Predisposition, Labile INR, Elderly, Drugs/Alcohol Concomitantly) scores [15, 16], where age is a significant factor due to the elevated bleeding risk in the elderly [17]. In our study, a robust statistical association in multivariate analysis was found between anticoagulation and adverse outcomes, including bleeding complications necessitating surgery and resulting in fatalities. This contrasts with the existing literature suggesting the safety of anticoagulant use. However, previous studies involved younger recipients, around 60 and 45 years of age, respectively [18, 19]. In our cohort, it is noteworthy that the predominant treatment among patients was anti-vitamin K therapy. Although direct oral anticoagulants have demonstrated non-inferiority in bleeding or mortality outcomes compared with warfarin and seem to be well-tolerated post-transplantation, there remains a gap in the literature regarding the assessment of complication rates associated with direct oral anticoagulation specifically in elderly kidney transplant recipients [20, 21].

This retrospective study has obvious limitations. Its retrospective design introduces potential biases. Although efforts were made to contact all centers involved in patient follow-up, there remains a risk of underestimating hospitalization duration in certain cases. Moreover, the absence of a control group, either of listed patients over 70 years or younger transplant patients, limit the interpretability of our findings. Despite analyzing numerous variables, only a few pertain specifically to geriatrics. This discrepancy arises from the historical lack of pre-transplantation frailty assessments by nephrologists. Several noteworthy tools have been developed and subjected to evaluation. One such tool is the Multidimensional Prognostic Index (MPI), which incorporates evaluations of functional, cognitive and nutritional status, as well as the risk of pressure sores, medication count and cohabitation status [22]. Evaluation of the MPI score revealed its efficacy in predicting outcomes such as all-cause mortality, number of hospitalizations and duration of hospital stays post-transplantation among patients aged 65 years and older [23]. The concept of frailty, along with its objective assessment, is a relatively recent development, gaining traction amid an increasing number of scientific publications [24, 25]. Lastly, the study does not delve into the notion of quality of life, representing another area that warrants future investigation.

In conclusion, the RETRAITE (REnal TRAnsplantIon ouTcome in Elderly recipients) study addresses some of the challenges associated with patient selection in kidney transplantation, especially for those aged 70 years and above. The study confirms that kidney transplantation in elderly patients can yield excellent results at 1 year, particularly in cases of preemptive transplants and those from living donors. Importantly, the recipient's age alone, in patients over 70 years, does not correlate statistically with higher morbidity and mortality. In addition to graft- and donor-related factors, the presence of curative anticoagulation emerges as a significant risk factor post-kidney transplantation in elderly recipients.

## Supplementary Material

sfae182_Supplemental_File

## Data Availability

The data underlying this article will be shared on reasonable request to the corresponding author.
