# Supplementary material for: Unraveling complexity: morbidity factors in elderly kidney transplant recipients
Source: Clin Kidney J. 2024 Jun 18;17(8):sfae182. doi: 10.1093/ckj/sfae182 (PMC11483501; doi:10.1093/ckj/sfae182)
Supplement: sfae182_Supplemental_File [file sfae182_Supplemental_File.docx]

Supplementary Table 1: Baseline characteristics of the 2 cohorts

|  | Kidney transplant recipients aged over 70 (n = 149) | Kidney transplant recipients aged between 60 -70  (n = 150) |
| --- | --- | --- |
| Male gender - n(%) | 105 (70) | 86 (57.3) |
| Age at transplantation – | 75.0 (3.6) | 64.3 (2.8) |
| Cold ischemia time - | 740 (387) | 754 (489) |
| High blood pressure – n (%) | 138 (93) | 125 (84.5) |
| Living donor | 25 (16.8) | 23 (15.3) |
| Dyslipidemia – n (%) | 88 (59) | 82 (54) |
| Smoking – n (%) | 77 (52) | 61 (40) |
| Diabetes – n (%) | 51 (34) | 44 (29.7) |
| Coronary heart disease – n (%) | 40 (27) | NA |
| Anticoagulation – n (%) | 35 (25) | 24 (16.0) |
| Peripheral arteriopathy – n (%) | 23 (15) | 14 (9.3) |
| Chronic respiratory disease – n (%) | 34 (23) | NA |
| Stroke or transient ischemic attack – n (%) | 15 (10) | NA |
| Chronic heart failure – n (%) | 8 (5) | 4 (6) |

Supplementary Table 2: Multivariate adjusted correlations with the composite endpoint during the first-year post-transplantation. Merged cohorts (study cohort, n=149 and control cohort n=150).

|  | Odd-ratio | 95% CI | p-value |
| --- | --- | --- | --- |
| Anticoagulation | **4.70** | **[2.27 – 9.97]** | **<0.001** |
| Peripheral arteriopathy | 2.18 | [0.88 – 5.2] | 0.084 |
| Dialysis before transplant | 1.35 | [0.26 – 10.0] | 0.740 |
| Time in dialysis before transplantation | 1.00 | [0.99 – 1.01] | 0.154 |
| Cold-ischemia time | 1.00 | [0.99 – 1.00] | 0.650 |
| **Donor age** | **1.05** | **[1.02 – 1.09]** | **0.002** |
| Deceased donor | 1.30 | [0.27 – 9.7] | 0.768 |
| **Delayed graft function** | **9.32** | **[4.00 – 23.1]** | **<0.001** |
| Basiliximab induction | 2.62 | [0.01 – 1.0] | 0.252 |

Supplementary Table 3: Unadjusted correlations with end-stage graft failure during the first-year post-transplantation.

|  | No graft loss  N= 142 | Graft loss within the 1^st^ year post KTx  N = 7 | p-value |
| --- | --- | --- | --- |
| Age at transplantation - Yr | 75.1 ± 3.6 | 72.9 ± 2.5 | 0.101 |
| Male gender - n(%) | 101 (71.1) | 4 (57.1) | 0.429 |
| High blood pressure – n (%) | 132 (93.0) | 6 (85.7) | 0.474 |
| Dyslipidemia – n (%) | 83 (58.5) | 5 (71.4) | 0.495 |
| Smoking – n (%) | 74 (52.1) | 3 (42.9) | 0.632 |
| Diabetes – n (%)  Insulin-treated – n (%) | 50 (35.2)  32 (22.5) | 1 (14.3)  1 (14.3) | 0.255  0.608 |
| Coronary heart disease – n (%) | 38 (26.8) | 2 (28.6) | 0.916 |
| Anticoagulation – n (%) | 34 (23.9) | 3 (42.9) | 0.258 |
| Peripheral arteriopathy – n (%)  With surgical intervention – n (%) | 22 (15.5)  11 (7.7) | 1 (14.3)  0 (0.0) | 0.931  0.444 |
| Chronic respiratory disease – n (%) | 33 (23.2) | 1 (14.3) | 0.582 |
| Stroke or transient ischemic attack – n (%) | 15 (10.6) | 0 (0.0) | 0.365 |
| Chronic heart failure – n (%) | 8 (5.6) | 0 (0.0) | 0.519 |
| Pre-emptive transplantation – n (%) | 18 (12.7) | 1 (14.3) | 0.992 |
| Duration time on the waiting-list - months | 33.7 ± 27 | 33.4 ± 24 | 0.982 |
| Cold-ischemia time - minutes | 735 ± 389 | 841 ± 363 | 0.302 |
| Donor age - Yr | 72.1 ± 10 | 72.7 ± 8.5 | 0.964 |
| Living donation– n (%) | 25 (17.6) | 0 (0.0) | 0.224 |
| Delayed graft function – n (%) | **19 (13.4)** | **6 (85.7)** | **< 0.001** |
| Induction therapy – n (%)  Anti-thymoglobulin  Basiliximab | 128 (90.1)  14 (9.9) | 7 (100.0)  0 (0.0) | 0.383 |

Supplementary Table 4: Unadjusted and adjusted (multivariate Cox analysis) correlation with cumulated prolonged hospitalizations above 40 days.

|  | Prolonged hospitalization (n=37) | Univariate p-value | Multivariate Odd-ratio, 95%IC | Multivariate p-value |
| --- | --- | --- | --- | --- |
| Age at transplantation - Yr | 75.1 (3.5) | 0.813 |  |  |
| Male gender – n (%) | 27 (73.0%) | 0.700 |  |  |
| High blood pressure – n (%) | 35 (94.6%) | 0.596 |  |  |
| Dyslipidemia – n (%) | 23 (62.2%) | 0.658 |  |  |
| Smoking – n (%) | 19 (51.4%) | 0.963 |  |  |
| Diabetes – n (%)  Insulin-treated – n (%) | 16 (43.2%)  12 (32.4%) | 0.183  0.082 |  |  |
| Coronary heart disease – n (%) | 13 (35.1%) | 0.189 |  |  |
| Anticoagulation – n (%) | **16 (43.2%)** | **0.003** | **2.9 (1.1 – 7.6)** | **0.027** |
| Peripheral arteriopathy – n (%)  With surgical intervention – n (%) | **11 (29.7%)**  **6 (16.2%)** | **0.006**  **0.018** | **4.1 (1.3 – 12.4)** | **0.011** |
| Chronic respiratory disease – n (%) | 11 (29.7%) | 0.248 |  |  |
| Stroke or transient ischemic attack – n (%) | 3 (8.1%) | 0.648 |  |  |
| Chronic heart failure – n (%) | 3 (8.1%) | 0.394 |  |  |
| Pre-emptive transplantation – n (%) | **1 (2.7%)** | **0.002** | 2.9 (4.1 – 58.3) | 0.360 |
| Duration time on the waiting-list - months | 40.2 (23.4) | 0.052 |  |  |
| Cold-ischemia time - minutes | **886.3 (288.2)** | **0.027** | 1.0 (0.9 – 1.0) | 0.771 |
| Donor age - Yr | **74.9 (8.8)** | **0.014** | 1. (0.1 – 1.1) | 0.235 |
| Living donation – n (%) | **1 (2.7%)** | **0.008** | 0.5 (0.02 – 4.8) | 0.559 |
| Delayed graft function – n (%) | **14 (37.8%)** | **< 0.001** | **6.4 (2.3 – 19.1)** | **< 0.001** |
| Induction therapy – n (%)  Anti-thymoglobulin  Basiliximab | 36 (97.3%)  1 (2.7%) | 0.108 |  |  |

Supplementary Figure 1: Comparison of the cumulative incidence of patient’s survival after kidney transplantation in 149 recipients over 70 years-old and 150 recipients between 60 and 70 years-oldn with and without anticoagulation treatment. Kaplan-Meier plots. P-values were calculated with the log-rank test.
